# Screening of additives in plastics with high resolution time-of-flight mass spectrometry and different ionization sources: direct probe injection (DIP)-APCI, LC-APCI, and LC-ion booster ESI

**DOI:** 10.1007/s00216-015-9238-5

**Published:** 2016-01-12

**Authors:** Ana Ballesteros-Gómez, Tim Jonkers, Adrian Covaci, Jacob de Boer

**Affiliations:** Institute for Environmental Studies, VU University Amsterdam, De Boelelaan 1087, 1081 HV Amsterdam, The Netherlands; Toxicological Center, University of Antwerp, Universiteitsplein 1, 2610 Antwerp, Wilrijk Belgium

**Keywords:** Plastics, Screening, Additives, Flame retardants, Mass spectrometry, Direct probe injection

## Abstract

Plastics are complex mixtures consisting of a polymer and additives with different physico-chemical properties. We developed a broad screening method to elucidate the nature of compounds present in plastics used in electrical/electronic equipment commonly found at homes (e.g., electrical adaptors, computer casings, heaters). The analysis was done by (a) solvent extraction followed by liquid chromatography coupled to high accuracy/resolution time-of-flight mass spectrometry (TOFMS) with different ionization sources or (b) direct analysis of the solid by ambient mass spectrometry high accuracy/resolution TOFMS. The different ionization methods showed different selectivity and sensitivity for the different compound classes and were complementary. A variety of antioxidants, phthalates, UV filters, and flame retardants were found in most samples. Furthermore, some recently reported impurities or degradation products derived from flame retardants were identified, such as hydroxylated triphenyl phosphate and tetrabromobisphenol A monoglycidyl ether.

**Electronic supplementary material:**

The online version of this article (doi:10.1007/s00216-015-9238-5) contains supplementary material, which is available to authorized users.

**Graphical abstract:**
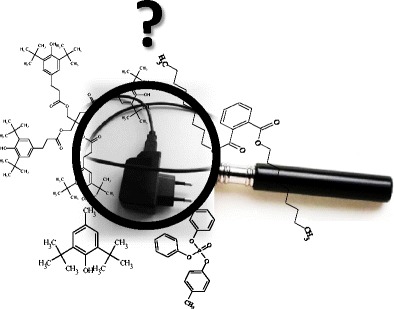
Wide screening of plastic additives by direct probe injection (DIP)-APCI, LC-APCI and LC-ion booster ESI

## Introduction

Due to their versatility and durability, plastics are very frequently used in consumer products. Additives, such as flame retardants, phthalates, antioxidants, or light stabilizers, are mixed with the plastic polymers for improving the final physical and chemical properties of the material. In general, flame retardants are added to prevent or minimize the spread of fire; phthalates enhance the flexibility, durability, and workability; and antioxidants and light stabilizers prevent or slow down the degradation. Some of these compounds have raised concern due to their ubiquity in the environment and their potential toxicity, e.g., some phthalates and UV filters have been reported to be endocrine disruptors [1, 2], and certain halogenated flame retardants have been reported to cause neurotoxicity and thyroid and sex hormone effects [3].

Due to the different chemical structures of the additives and the plastic matrix, the analysis of additives in plastics is a challenging task. Methods based on solvent extraction and liquid chromatography-mass spectrometry (LC-MS) [4–7], pyrolysis gas chromatography (GC)-MS [8], and capillary electrophoresis (CE)-MS [9] have been used before. Recently, ambient mass spectrometry has been proposed as a suitable alternative for screening additives in plastics since it does not require any sample preparation [10, 11]. The option of analyzing solid material in its native state without any extraction or clean-up can provide information that is complementary to that generated by conventional methods, although sometimes the complex mass spectrum generated can be difficult to interpret. A variety of ambient mass ionization techniques have been proposed for the analysis of polymers and additives, such as direct-analysis-in-real-time (DART), desorption electrospray ionization (DESI), atmospheric pressure ionization (APCI) solid analysis probe (ASAP), or atmospheric pressure ionization-matrix-assisted laser desorption/ionization (AP-MALDI) mostly combined with high resolution mass spectrometry (HRMS) [10, 11]. In general, the reported methods were applied to certain types of additives or to a selected number of compounds from different classes. However, wider and yet simple screening techniques for additives would be interesting for quality control and also for future toxicity studies.

The plastic polymers as such are considered inert. The potential toxicity of plastics is caused by additives that are suspected to leach out into the environment and, dependent on the dose, cause health problems as mentioned before. Plastics are also one of the most common and persistent debris in ocean waters and beaches worldwide [12]. Plastic leachates in water have been reported to exert aquatic toxicity with *Daphnia magna* [13] and *Nitocra spinipes* [14], depending of the type of plastic and the weathering conditions. Furthermore, the migration of certain potentially harmful components from plastics to contact media has also been investigated, e.g., bisphenol A from polycarbonate baby bottles [15] or flame retardants from plastics of electronic products [16].

In this study, we aimed to characterize a wide variety of potentially toxic plastic additives used in casings of electronic/electrical appliances. Solvent extracts and solids were analyzed by LC-high resolution/accuracy-time-of-flight (TOF) MS with different ionization sources or by ambient mass spectrometry with direct probe (DP)-APCI, respectively. These techniques proved to be complementary providing with different selectivity and sensitivity for the different compounds classes. Some novel flame retardants and related impurities, for which data in the literature is very limited, were identified in many samples.

## Experimental section

### Chemical and reagents

Acetone and methanol (MeOH) were from J.T. Baker® (Center Valley, USA). Tetrahydrofuran (THF) was acquired from Biosolve (Valkenswaard, The Netherlands). Dichloromethane (Picograde) was obtained from Promochem® (Wesel, Germany) and toluene came from Fisher Scientific (Loughborough, UK). Milli-Q water was obtained from ultrapure water purification Q-Pod system (Millipore, Bedford, USA). All solvents and reagents were of analytical grade and used as supplied.

For sample treatment, micro-centrifuge filters (0.2 μm, nylon) from Costar Spin-X obtained from Sigma-Aldrich were used for removing micro-particles from sample extracts when necessary.

### Apparatus

A microTOF II with resolution >16,500 FWHM was used as detector and equipped with an LC-APCI II, direct probe-APCI or an LC-electrospray ionization (ESI)-ion booster source (Bruker Daltonics, Bremen, Germany). For LC, an InertSustain C18 (3 μm particle size, 10 mm length) precolumn and an InertSustain C18 (3 μm particle size, 2.1 mm i.d., 100 mm length) column were used as stationary phase (GL Sciences, Eindhoven, The Netherlands). For the mobile phase, Milli-Q water and MeOH were used in the following gradient: 50 % MeOH (*v*/*v*) for 0.5 min, a linear gradient to 98 % MeOH (*v*/*v*) in 15 min followed by 98 % MeOH (*v*/*v*) for 10 min. The flow was 0.3 mL/min, the column temperature was set at 30 °C, and the injection volume was 5 μL.

### Sample collection and preparation

Electrical/electronic devices (*n* = 28) were bought in supermarkets in the Netherlands in 2014 and 2015 (e.g., hair irons, keyboards, routers, PC loudspeakers, USB phone chargers) or collected directly from Dutch houses and/or offices. Small amounts of material (10–20 mg) were scratched off (e.g., from televisions, dryer, printers). A detailed list of the samples is given in Electronic Supplementary Material Table S1. Although we did not determine the polymer present in the samples, we expect a variety of different polymer plastics, being high-impact polystyrene (HIP), acrylonitrile butadiene styrene (ABS), polypropylene (PP), polyurethane (PU), or polycarbonate (PC) very common among these type of samples.

Plastic subsamples (*n* = 50) from the electronic/electrical devices (mainly plastic casings) were taken using a surgical cutter and a Stanley knife. If a device contained different sorts of plastic, these were sampled individually. Samples (10 mg) were extracted with 1.5 mL of a mixture THF:MeOH (70:30, *v*/*v*) by sonicating (30 min) and stirring (200 rpm) for 12 h. Extracts were diluted 1:1 with MeOH, ultra-centrifuged, and further filtrated if required (with 0.2 μm micro-centrifuge filters). Aliquots of 5 μL were further analyzed by LC-MS. In order to cover a wide range of compounds, no clean-up was performed before analysis.

### Sample analysis

For the calibration of the TOF-HRMS, a solution forming sodium formate adducts was used with the LC-ESI-ion booster and a commercial calibration solution with the APCI source (APCI-LC low concentration tuning mix, Agilent Technologies) for both direct probe or LC-APCI measurements. All samples were analyzed in the positive and negative mode. Calibration was done before each batch of experiments or in each sample (within the first minute of the chromatogram) by means of a syringe pump and a switching valve in LC of by adding a drop of calibrant directly onto the probe.

Some MS-TOF parameters are very dependent on the *m*/*z* value and can influence the sensitivity very much. These parameters were optimized for a range of *m*/*z* of 100–1200. Capillary exit and skimmer 1 were set at 90 and 30 V, hexapole RF at 250 Vpp, transfer time at 50 μs, and pulse storage time at 10 μs. The heater temperature was set at 220 °C and the vaporizer temperature in LC measurements at 280 °C. For the direct probe, a gradient of vaporizer temperature was set in order to gradually elute different compounds from the matrix and to obtain in this way some degree of selectivity. The vaporizer temperature was increased from 200 to 250 °C after 30 s and increased again to a temperature of 320 °C after 90 s and then hold at this temperature for another 60 s. Higher temperatures were avoided to prevent vaporization/ionization of the plastic polymers that would mask the signal of the additives in the mass spectra. The temperature of the vaporizer was, however, increased between samples, once the loaded probe was removed from the source, till 350–400 °C for 5–20 min in order to clean the source. Blanks (unloaded samples) were run between samples to check for possible cross-contamination.

### Data processing

The software *data analysis* and *target analysis* from Bruker Daltonics (Bremen, Germany) was used for data processing. Confirmation of the presence of analytes was based on mSigma values (match factor between the measured isotopic pattern and the theoretical pattern for a given formula) and mass accuracy. Values of less than 5 ppm of mass error and less than 100 of mSigma were considered acceptable for positive confirmation (mSigma <100 acceptable, <50 good, and <25 excellent).

### Targeted screening of additives

An in-house database of around 250 compounds was used for target screening based on the molecular formula. Additives were compiled from literature and from commercial catalogs. The ions [M-H]^−^, [M+H]^+^, and [M+Na]^+^ were considered the most probable together with [M-Br+O]^−^, [M-Cl+O]^−^, [M+O_2_]^−^ for brominated compounds in APCI(-) as described before [17]. Compounds were tentatively identified based on mass error and isotopic pattern fit.

A group of flame retardants (*n* = 22) were injected as authentic standards and their retention times and calculated log K_ow_ used as a reference to the predict retention times along the LC gradient and to confirm in this way the identity of the compounds. Acceptable time windows of ±3 and ±5 min were set for compounds with retention times below and above or equal to 17.5 min, respectively.

Highly brominated compounds (more than five or six bromine atoms) were not recognized by target analysis/database approach due to limitations of the software with respect to the isotopic pattern fit. For these additives, the tool isotope cluster analysis of the software data analysis of Bruker was used to identify possible matches in LC measurements [18]. This tool searches for chemicals with a noticeable isotopic pattern and set up chromatograms by summing up the intensities of isotopomer peak pairs of specified *m*/*z* difference and intensity ratio values. The *m*/*z* difference was set at 2 and the intensity ratios for molecules with different number of Cl or Br atoms can be found in reference 18.

Since additives are ubiquitous in many laboratory materials, procedural blanks were routinely run with each batch of samples. Low levels of some additives, mainly cyanox 1790 (CAS n° 40601-76-1), bis(2-ethylhexyl) phthalate (DEHP), di-isononyl phthalate (DINP), and Irganox 1076 (CAS n° 2082-79-3), were found in LC procedurals blanks, while low levels of DEHP, DINP, and dibutyl phthalate (DBP) were found in direct probe blanks. These contamination levels were taken into account as noise to calculate the detection limits of the techniques.

A final visual inspection of the positive matches was done to prevent false positives (e.g., compounds coming from in-source fragmentation of bigger molecules).

The following criteria were taken into account to identify a positive peak: (a) the mass accuracy (ppm error) was below 5 ppm and the isotopic pattern fit (msigma value) below 100 msigma; (b) the signal was at least of 5000 counts of intensity or three times higher than the background noise in blanks; (c) the retention time was the same (±0.2 min) in all samples and was consistent with the predicted retention time based on the calculated log K_ow_ (±3 and ±5 min for retention times <17.5 or ≥17.5 min, respectively); (d) the ionization mode giving a higher response was consistent with the chemical structure of the compound (negative and/or positive and ESI or APCI); and (e) the matches were not false (e.g., they do not come from in-source fragmentation peaks of bigger molecules).

### Untargeted screening of flame retardants

Since electrical/electronic equipment usually contains flame retardants to comply with flammability standards, special attention was given to this group of additives. Untargeted screening was done to identify possible impurities and degradation products of the most abundant flame retardants by using the tool smart formula of the data analysis program to generate formulae. General parameters for formulae generation were as follows: mass error tolerance was set to 5 ppm; H/C ratio from 0 to 3; number of rings and double bonds was restricted from 0.5 to 40. The option automatically detect isotopic mass was activated when looking for halogenated compounds. A detailed data processing flowchart for the identification of the halogenated compounds can be found in reference 18.

## Results and discussion

### Solvent leaching step for the screening of additives in plastics

Accelerated solvent extraction [19, 20] and microwave-assisted extraction [21] have been used in the literature for the extraction of additives from plastics. In this study, we investigated a liquid-solid extraction based on sonication and stirring to leach out the main additives from the plastics as a simpler procedure for screening purposes. The use of sonication as extraction method for flame retardants from plastics has been proposed before [22–24].

**Fig. 1:**
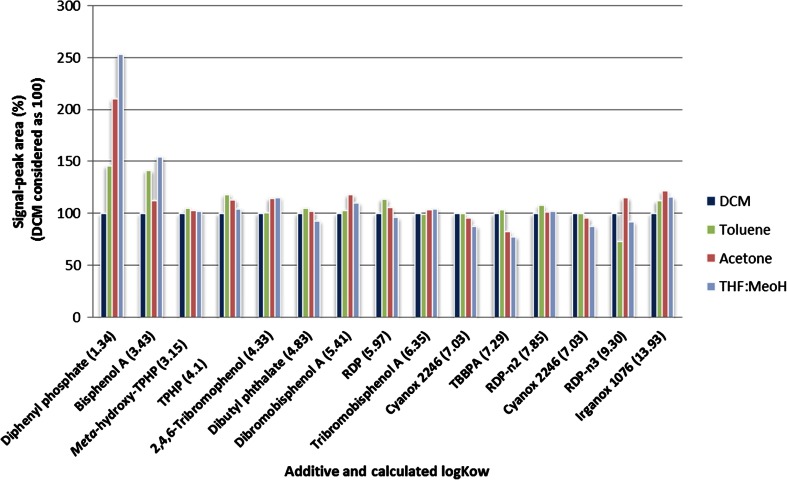
Extraction of additives with different solvents from two plastic samples (sample 25-printer and sample 19-router). Values are shown as peak area signal that is normalized to the results obtained with dichloromethane (considered as 100 %). Values are an average of three independent experiments. Calculated log K_ow_ values are shown in brackets (ACD/ChemSketch, ACD/Labs). *TBBPA* tetrabromobisphenol A, *TPHP* triphenyl phosphate, *RDP* resorcinol bis(diphenyl phosphate), *RDP-n2* resorcinol bis(diphenyl phosphate) dimer, *RDP-n3* resorcinol bis(diphenyl phosphate) trimer, *DCM* dichloromethane

Different solvents were used to assure the screening of a wide polarity range of compounds. Two plastic samples containing additives with a wide polarity range (calculated log K_ow_ 1.34–13.93) were selected for optimization of the leaching solvent for screening. Since plastics are homogeneous, aliquots of 10 mg were used in order to scale down the procedure and to minimize in this way the use of solvents and materials. Different extraction solvents were tested (1.5 mL of acetone, toluene, dichloromethane, or of a mixture THF:MeOH 30:70 *v*/*v*). Figure 1 shows the signal of each compound as peak area (average of three experiments, normalized to the signal of dichloromethane that was considered as 100 %). Despite the different polarity range between the additives, we did not find significant differences between the different solvents, except for the more polar ones (bisphenol A and diphenyl phosphate) that were better extracted in the most polar solvents acetone and THF:MeOH. Since THF:MeOH gave good results and is a water-soluble solvent, making the compatibility with liquid chromatography easier, it was selected as optimal solvent for further screening experiments. No further clean-up was made in order to prevent losses of compounds.

### MS ionization sources for screening additives in plastics

**Fig. 2:**
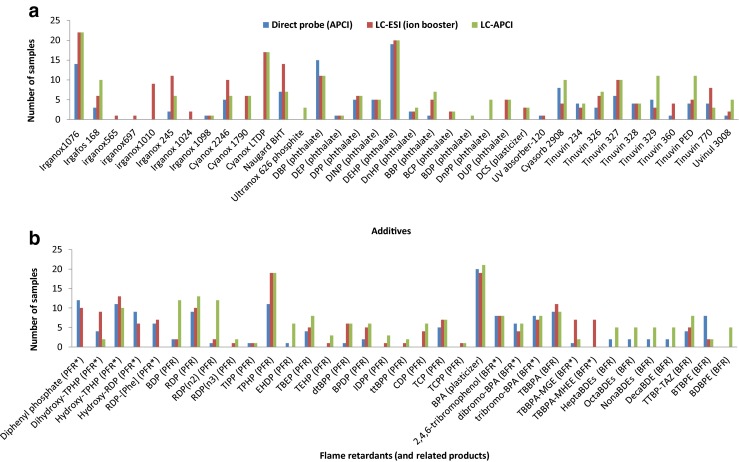
**a** Additives and **b** flame retardants and related products that were identified in plastic samples from electrical/electronic products by different ionization mechanisms (direct probe-APCI, LC-ESI, and LC-APCI) Abbreviations: Phosphorus flame retardants (PFR) and related compouunds (PFR*), brominated flame retardants (BFR) and related compounds (BFR*). The full chemical names and structures of the compounds are given in Table S-2

A variety of additives (*n* = 71) including antioxidants (*n* = 13), phthalates (*n* = 11), non-phthalates plasticizers (*n* = 2), light stabilizers (*n* = 11), and flame retardants and related products (*n* = 34) were detected in the electrical/electronic products as shown in Fig. 2. A detailed list of the additives detected with their molecular formula, structure, and main ion measured in each ionization mode is given in Electronic Supplementary Material Table S2. The main ions observed for these compounds were [M-H]^−^ and [M+H]+ in both ESI and APCI (LC and direct probe mode), except for phosphorus flame retardants (PFRs), phthalates, and antioxidants from the Cyanox class (hindered phenolic orthoester-based compounds) that were detected as [M+Na]^+^ in ESI (+). Other exceptions were polybrominated diphenyl ethers (PBDEs) and highly brominated flame retardants, that were detected as [M-Br+O]^−^ in APCI(-) and BTBPE [1,2-bis(2,4,6-tribromophenoxy)ethane] detected in both ESI(-) and APCI(-) as a fragment [C_6_Br_3_H_2_O]^−^. Other secondary ions were observed, such as [M+CH_3_OH+H]^+^ and [M+CH_3_CNNH_3_+H]^+^ in APCI(+) for some PFRs.

The sensitivity and selectivity can differ between the ionization methods and between the different samples matrices and this caused some variability between the compounds detected with each method, mainly when the additives where present at low levels. LC-APCI was more selective than LC-ESI and more sensitive for non-polar compounds that are difficult to ionize, even given the fact that the ESI-ion booster source counts with an additional soft voltage and a vaporizer temperature for enhancing the ionization. For this reason, PBDEs and decabromodiphenyl ethane (DBDPE) were only detected in APCI mode. For other non-polar compounds, although they were detected by both LC-ESI and LC-APCI, the number of positive samples was higher when using LC-APCI because of its better sensitivity, e.g., tris(2,4,6-tribromophenoxy)-1,3,5-triazine (TTBP-TAZ), bisphenol A bis(diphenyl phosphate) (BDP or BPA-BDPP), or the dimer and trimer of resorcinol bis(diphenyl phosphate) (RDP or PBDPP). The LC-APCI method was also advantageous in terms of lower background noise. This resulted in lower limits of detection for some compounds even if similar absolute peak intensities were observed for both ESI and APCI modes. Consequently, the LC-APCI method gave more positive matches for some PFRs, such as di-tert-butylphenyl phenyl phosphate or 2-ethylhexyl diphenyl phosphate (EHDP), phthalates like di-*n*-propyl phthalate (DnPP), and light stabilizers from the Tinuvin class (hindered amine derivatives, e.g., Tinuvin 329 and Tinuvin PED). The ESI-ion booster source in positive mode suffered also from masking signals of high intensities coming from polymers. For this reason, when using ESI, the additives from the Tinuvin class were mainly only identified in negative mode while they were detected as both [M-H]^−^ and [M+H]^+^ in APCI (see Electronic Supplementary Material Table S2).

On the other hand, a higher number of positives were found by the LC-ESI method for some compounds detected at low levels for which this source was more sensitive, such as impurities of TBBPA derivatives, namely tetrabromobisphenol A mono(2-hydroxyethyl ether) (TBBPA-MHEE) and tetrabromobisphenol A mono(glycidyl ether) (TBBPA-MGE) and compounds from the Irganox class, like Irganox 1010. In fact, better sensitivity (ten times better) has been reported for Irganox 1010 for ESI compared to APCI [25]. Furthermore, the most polar compounds, such as diphenyl phosphate, were mainly detected in ESI.

Finally, the direct probe-APCI method (without sample preparation or chromatography) was applicable to every compound class. Results were in agreement with those obtained by LC-APCI, but only high levels could be detected (estimated limits above 0.1 % in weight were calculated for flame retardants) [17]. A disadvantage of the direct probe was the presence of false positives due to in-source fragmentation. For example, BTBPE could be misidentified with 2,4,6-tribromophenol, since the fragment monitored for the first one shares the same molecular formula than the second one. False positives were also caused for diphenyl phosphate and some hydroxylated PFRs, which could be generated by in-source fragmentation or displacement reactions with oxygen, respectively. Despite these limitations, the direct probe method is very simple and rapid and can also enhance the detection of compounds in a complex matrix with low solvent extraction recoveries. This method is also very useful for a first pre-screening of samples at high levels of additives prior to LC analysis and to save time and consumables.

In general, for broad screening, the combination of complementary ionization methods is very useful to cover a wide range of compounds.

### Retention time prediction

**Fig. 3:**
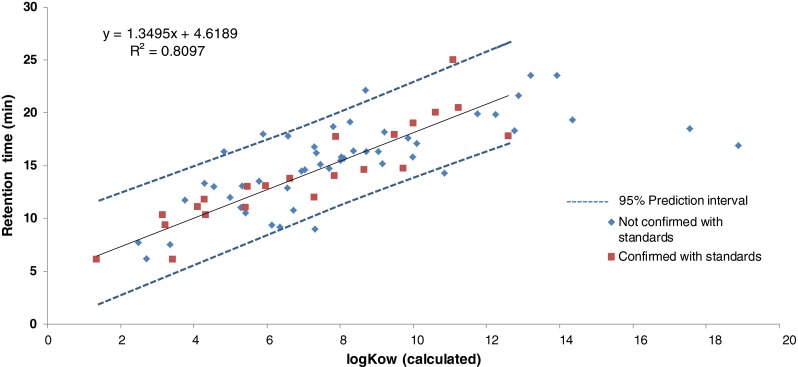
Retention times of the identified compounds vs. calculated log Kow (ACD/ChemSketch, ACD/Lab). Compounds that were confirmed with the injection of the authentic standards are in *red*, while those not confirmed are in *blue*

In wide-scope screening methods, LC retention time prediction can be useful for confirmation of the tentatively identified compounds. Although there are many commercial in silico programs available for the prediction of retention times that are based on various molecular descriptors, the use of only log K_ow_ has been reported as a simple successful approach [26, 27]. As can be seen in Fig. 3, the correlation between the retention time and the calculated log K_ow_ of the injected standards was high (*R*^2^ = 0.8097, *n* = 22). Around 80 % of the retention times of the identified compounds were predicted with a ±3 min time window and 100 % with a ±5.0 min. Around 45 and 65 % of the compounds were predicted with time windows of ±1 and ±2 min, respectively. Similar results were found by Bade et al. [27], with windows of ±2 min for 70 % of compounds and of ±4 min for 95 % of the compounds by using a UPLC program of 16 min.

In this study, the compounds with a higher deviation in their predicted retention times (±5.0 min) all eluted later in the chromatogram (≥17.7 min), after the LC linear gradient reached a constant value of 98 % MeOH, so that a certain flattening of the trend would be expected. Therefore, retention times window for confirmation based on the predicted retention times were set at ±3 and ±5 min for compounds with measured retention times below or equal/above to 17.5 min. In this way, a common cause of false positives, in-source fragmentation, could be avoided. In addition to the aforementioned possible misidentification of 2,4,6-tribromophenol, diphenyl phosphate could also be falsely detected as a fragment coming from triphenyl phosphate (TPHP) or from resorcinol diphenyl phosphate (RDP).

Only two compounds with very high calculated/predicted values of log K_ow_ (17.56 for Irgafos 168 and 18.89 for Irganox 1010) did not comply with the acceptable time window, but this is probably due to a deviation of the calculated log K_ow_ from the experimental value, occurring more often in complex high molecular weight molecules.

### Presence of flame retardants, plasticizers, impurities and degradation products

Samples of electrical/electronic equipment frequently contain flame retardants, so we further investigated this group of additives. Out of 28 casings of electrical or electronic plastic equipment, 16 contained flame retardants. Flame retardants were detected in a total of 20 samples coming from these products.

Brominated flame retardants (BFRs) were present in 11 samples and contained a mix of them instead of a single flame retardant as we already reported before [28]. The presence of a mix of flame retardants may be due to synergistic reasons, but could also be caused by cross-contamination in the factory or during recycling or. TBBPA (*n* = 11), 2,4,6-tribromophenol (*n* = 8), PBDEs (*n* = 5), TTBP-TAZ (*n* = 8), BTBPE (*n* = 2), and BDBPE (*n* = 5) were detected. In terms of absolute intensity, TBBPA was the major flame retardant in most samples containing BFRs.

Regarding PFRs, TPHP was present in almost all samples (*n* = 19) containing flame retardants. So, PFRs and BFRs were often both present in the tested samples. RDP and BDP were also frequently detected (*n* = 13 and 12, respectively). In fact, TPHP is present in RDP and BDP formulations (1–5 % *w*/*w*) as impurity. RDP was one of the most abundant flame retardants in terms of absolute intensity. Other PFRs or related plasticizers were also frequently detected, such as tris(2-butoxyethyl) phosphate (TBEP) (*n* = 8), di-tert-butylphenyl phosphate (*n* = 6), tert-butylphenyl diphenyl phosphate (BPDP) (*n* = 6), cresyl diphenyl phosphate (CDP) (*n* = 6), and tricresyl phosphate (TCP or TMPP) (*n* = 7).

Besides flame retardants and related plasticizers, samples can contain oligomers, degradation products that could be formed during the processing or aging of the plastics and byproducts, this increasing the complexity of the matrix. Since TBBPA and RDP were the most abundant flame retardants in the samples, we screened for possible related compounds by an untargeted approach. TBBPA-MGE (TBBPA monoglycidyl ether) and TBBPA-MHEE (TBBPA mono 2-hydroxyethyl ether), two impurities recently reported in the literature [29], were detected in 7 of the 11 samples that contained TBBPA. Also, di- and tribromobisphenol A as debromination/degradation products of TBBPA were detected in 6 and 8 samples, respectively. Bisphenol A was also present in 10 of the 11 samples that contained TBBPA, but since it is widely used as plastic monomer, it is difficult to relate it exclusively to TBBPA.

**Fig. 4:**
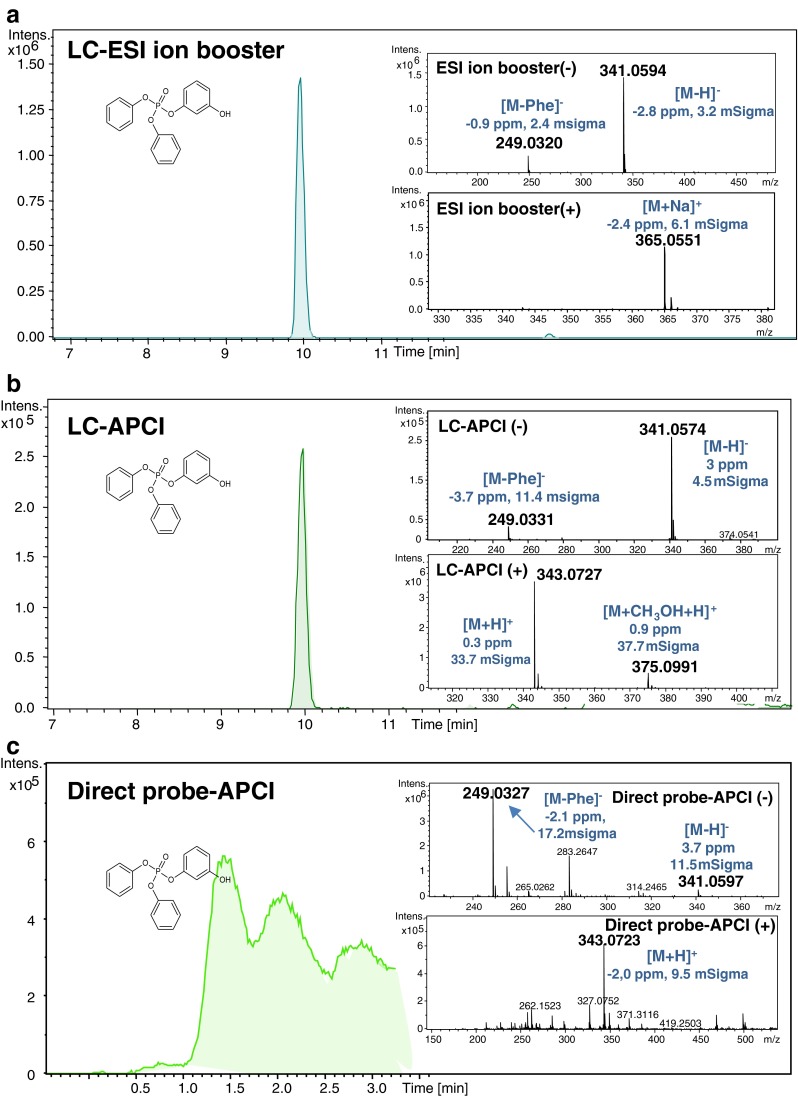
Identification of *meta*-hydroxy-triphenyl phosphate (RDP impurity) in a printer plastic sample. The extracted ion chromatogram of [M-H]^−^ for LC-ESI, LC-APCI, and direct probe-APCI are shown in **a**, **b**, and **c**, respectively. The spectrum (in negative and in positive mode) with the major ions of the target compound and their mass error values and isotopic pattern fit (or mSigma value) are displayed as an insert in each analysis method. The ion corresponding to diphenyl phosphate that was coming from the in-source fragmentation of the target compound in LC and also from structurally related compounds in direct probe-APCI is also shown

In samples with high levels of RDP, a variety of impurities and or breakdown products coming from hydrolysis and/or oxidation were detected, namely diphenyl phosphate, hydroxylated triphenyl phosphate, hydroxylated RDP, and RDP with the loss of phenyl group (RDP-[Phe]). These RDP-related products were recently reported by our group in vitro metabolism and hydrolysis experiments [30]. As an example, the Fig. 4 shows the chromatograms (or signal vs. intensity) and major ions monitored for the identification of *meta*-hydroxy-triphenyl phosphate by the three analysis methods. The dimer and trimer of RDP were also detected in 12 and 2 samples, respectively, and most favorably with the APCI source that is more sensitive for non-polar compounds.

For these impurities or degradation products of RDP and TBBPA derivatives, the potential toxicity and biodegradability was estimated with the free in silico program Toxtree (http://toxtree.sourceforge.net/). To the best of our knowledge, there is no data in the literature or chemical databases about the toxicity of these compounds. Results are shown in Electronic Supplementary Material Table S3. The program estimate toxic hazard by applying a decision tree approach based mainly on the chemical structure of the molecule. For the toxicity evaluation, the decision tree “Cramer rules with extension” was applied. Under this option, chemicals are divided into three structural classes according with concern for their potential toxicity: class I (low), class II (intermediate), and class III (high). All compounds, except for diphenyl phosphate (class I, organophosphates negatively charged), were classified as class III. Class III includes substances that permit no strong initial presumption of safety, or may even suggest significant toxicity or have reactive functional groups, while class I substances are simple chemical structures with efficient modes of metabolism suggesting a low order of oral toxicity. The decision tree “START biodegradability” was also applied to predict the biodegradability/persistency. All compounds were classified as persistent because of the presence of at least two benzene rings. Finally, also mutagenicity and carcinogenicity decision trees were applied and alerts were only found for TBBPA-MHEE (impurity of TBBPA derivatives), due to presence of an epoxy group.

Toxicity and biodegradability prediction can greatly differ from experimental results since the rules are only based on the chemical structure of the compounds and are very restrictive. In general, many of these impurities or degradation products that seem to be ubiquitous in electrical/electronic devices and may leach to the environment have been scarcely or not studied at all in environmental samples, such as indoor dust, and this would be desirable for a better assessment of the exposure to flame retardants. Furthermore, since most of these substances are estimated as of high concern, future biological testing is also necessary for the determination of a possible risk to human health.

On the basis of these results, more environmental data are also needed for alternative flame retardants recently reported in indoor samples such as RDP and BDP [31] and TTBP-TAZ [28] and some PFRs also frequently detected in the samples of electrical/electronic products.

## Conclusions

- A broad screening approach based on a simple solvent leaching step and the combination of different ionization method (LC-ESI-ion booster, LC-APCI, direct probe-APCI) coupled with a high resolution TOF-MS and time retention prediction based on log Kow is proposed for the screening of additives in plastics, in order to obtain a complete overview of the different compounds classes present in the samples, both by targeted and untargeted data processing. While LC-ESI-ion booster was needed to ionize the most polar compounds, such highly polar PFRs, APCI was necessary for the detection of very non-polar additives, such as PBDEs. On the other hand, the direct analysis of solids by ambient mass spectrometry was simple, rapid, and could enhance the detection of compounds in complex matrices with low solvent extraction recoveries. Although the identification by this technique is limited by the lack of chromatographic separation and the presence of false positives due to in-source fragmentation, it could be used as a fast and inexpensive pre-screening technique with a wide applicability.
- A variety of antioxidants, light stabilizers, plasticizers, and flame retardants (*n* = 71) were identified in plastic from electrical/electronic products. Alternative flame retardants (RDP, BDP, TTBP-TAZ) and degradation products or impurities of flame retardants (related to RDP and TBBPA derivatives) that have been scarcely or not previously reported were identified in this study and they could be interesting for future environmental studies assessing the human exposure to flame retardants.

## Electronic supplementary material

Below is the link to the electronic supplementary material.

ESM 1 (PDF 666 kb)

## References

[1] Peijnenburg WJGM. Phthalates. Reference module in earth systems and environmental sciences, encyclopedia of ecology. 2008. p. 2733–38.

[2] Lyche JL, Rosseland C, Berge G, Polder A (2015). Environ Int.

[3] Ponzo OJ, Silivia C (2013). Toxicology.

[4] Reingruber E, Himmelsbach M, Sauer C, Buchberger W (2010). Polym Degrad Stab.

[5] Noguerol-Cal R, Lopez-Vilarino JM, Gonzalez-Rodriguez MV, Barral-Losada LF (2007). J Sep Sci.

[6] Gaudin K, Ho-Sung H, Bleton J, Joseph-Charles J, Dallet P, Puig P (2007). J Chromatogr A.

[7] Gill M, Garber MJ, Yousheng H, Jenke DJ (2010). Chromatogr Sci.

[8] Hintersteiner I, Schmid T, Himmelsbach M, Klampfl CW, Buchberger WW (2014). Electrophoresis.

[9] Fries E, Dekiff JH, Willmeyer J, Nuelle MT, Ebert M, Remy D (2013). Environ Sci Processes Impacts.

[10] Yang R, Zhao JH, Liu Y, Rui Y (2013). Polym Degrad Stab.

[11] Paine MRL, Barker PJ, Blanksby SJ (2014). Anal Chim Acta.

[12] Moore CJ (2003). Nat Hist.

[13] Bejgarna S, MacLeoda M, Bogdala C, Breitholtza M (2015). Chemosphere.

[14] Maragou NC, Makri A, Lampi EN, Thomaidis NS, Koupparis MA (2008). Food Addit Contam.

[15] Kim YJ, Osako M, Sakai SI (2006). Chemosphere.

[16] Llompart M, Sanchez-Prado L, Lamas JP, Garcia-Jares C, Roca E, Dagnac T (2013). Chemosphere.

[17] Ballesteros-Gómez A, de Boer J, Leonards PEG (2013). Anal Chem.

[18] Ionas AC, Ballesteros-Gómez A, Leonards PEG, Covaci A (2015). J Mass Spectrom.

[19] Zhang Y, Du Z, Li A, Tu A, Yuc W, Zoud (2013). J Anal Methods.

[20] Li B, Wang Z-W, Lin QB, Hu CY, Su QZ, Wu YM (2015). J Chromatogr Sci.

[21] Sternbauer L, Dieplinger J, Buchberger W, Marosits E (2014). Talanta.

[22] Ionas AC, Dirtu AC, Anthonissen T, Neels H, Covaci (2014). Environ Int.

[23] Kajiwara N, Noma Y, Takigami HJ (2011). Hazard Mater.

[24] Ballesteros-Gómez A, Brandsma SH, de Boer J, Leonards PEG (2013). Chemosphere.

[25] Pouech C, Lafay F, Wiest L, Baudot R, Léonard D, Cren-Olivé C (2014). Anal Bioanal Chem.

[26] Nurmi J, Pellinen AL, Rantalainen (2012). J Mass Spectrom.

[27] Bade R, Bijlsma L, Sancho JV, Hernández F (2015). Talanta.

[28] Ballesteros-Gómez A, de Boer J, Leonards PEG (2014). Environ Sci Technol.

[29] Liu A, Qu G, Zhang C, Gao Y, Shi J, Du Y (2015). J Chromatogr A.

[30] Ballesteros-Gómez A, Van den Eede N, Covaci A (2015). Environ Sci Technol.

[31] Brandsma SH, Sellstrom U, de Wit CA, de Boer J, Leonards PEG (2013). Environ Sci Technol.

