# Supplementary material for: Screening of additives in plastics with high resolution time-of-flight mass spectrometry and different ionization sources: direct probe injection (DIP)-APCI, LC-APCI, and LC-ion booster ESI
Source: Anal Bioanal Chem. 2016 Jan 12;408:2945–53. doi: 10.1007/s00216-015-9238-5 (PMC4819935; doi:10.1007/s00216-015-9238-5)
Supplement: Supplementary file 1 — (PDF 666 kb) [file 216_2015_9238_MOESM1_ESM.pdf]

Analytical and Bioanalytical Chemistry

Electronic Supplementary Material

Screening of additives in plastics with high resolution time-of-flight mass spectrometry and different ionization sources: direct probe injection (DIP)-APCI, LC-APCI, and LC-ion booster ESI

Ana Ballesteros-Gómez, Tim Jonkers, Adrian Covaci, Jacob de Boer

Table S-1. Samples and subsamples of electrical/electronic products.

Sample	Product	Product/subsamples
1	Dust collector	Body
2	Dryer	Body
3	Heat sealer	body
4	Mobile phone charger A	Body, cable, plug
5	MP3 charger	Body 1, body 2
6	Mobile phone car charger	Body 1, body 2
7	(Land) phone	Body
8	Loudspeaker	Body
9	Electrical powerboard A	Body
10	Electrical powerboard B	Body
11	Electrical powerboard C	Body
12	Television A	Body
13	Television B	Body
14	Television C	Body
15	Hair Straightener A	Body 1, body 2, cable
16	Hair Straightener B	Body 1, cable, plug
17	Hair Straightener C	Body
18	Hair curling iron	Body 1, body 2, cable, plug
19	Router	Body 1, body 2
20	Computer keyboard A	Body 1, body 2, cable
21	Computer keyboard B	Body 1, body 2, cable
22	Computer	Body 1, body 2
23	Computer mouse	Body 1, body 2, plug
24	Printer A	Body
25	Printer B	Body
26	Christmas light A	Body, cable
27	Christmas light B	Body
28	Heater (fan) A	Body 1, propeller, cable

Table S-2. Additives identified in plastics from electrical/electronic products (name, class, formula, structure, number of positive samples and measured ions)

Additive (common name)	Additive class	CAS number	Formula	Structure	Number of samples[a]	Measured ions
Irganox1076	Antioxidant	2082-79-3	C35H62O3	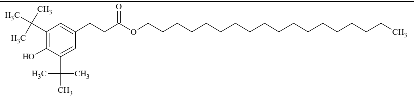	22	LC-ESI: [M+H]$^+$, [M-H]$^-$<br>LC-APCI: [M+H]$^+$, [M-H]$^-$<br>DIP-APCI: [M-H]$^-$
Irgafos 168	Antioxidant	31570-04-4	C42H63O3P	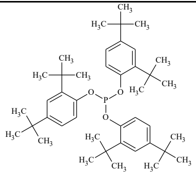	10	LC-ESI: [M+H]$^+$,<br>LC-APCI: [M+H]$^+$<br>DIP-APCI: [M+H]$^+$
Irganox 565	Antioxidant	991-84-4	C33H56N4OS2	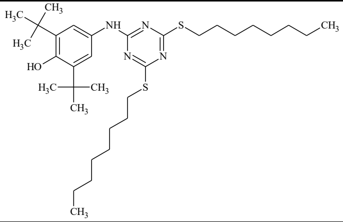	1	LC-ESI: [M+H]+, [M-H]-
Irganox 697	Antioxidant	70331-94-1	C40H60N2O8	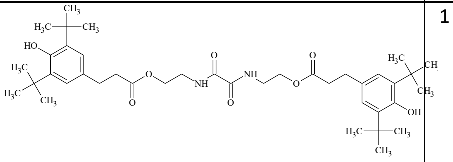	1	LC-ESI: [M+H]+
Irganox 1010	Antioxidant	6683-19-8	C73H108O12	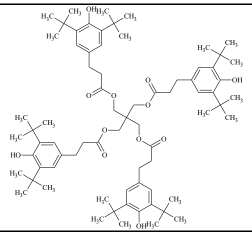	9	LC-ESI: [M+Na]+, [M-H]-
Irganox 245	Antioxidant	36443-68-2	C34H50O8	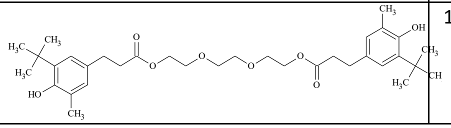	11	LC-ESI: [M+Na]+, [M-H]-<br>LC-APCI: [M+H]$^+$, [M-H]$^-$<br>DIP-APCI: [M-H]$^-$

Irganox 1024	Antioxidant	32687-78-8	C34H52N2O4	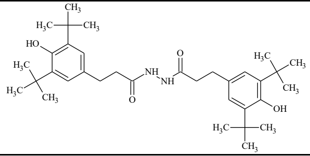	2	
Irganox 1098	Antioxidant	23128-74-7	C40H64N2O4	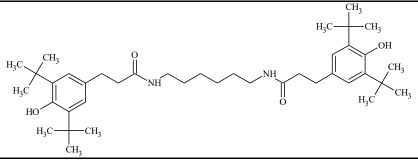	1	LC-ESI: [M+Na]+, [M-H]-<br>LC-APCI: [M+H]+<br>DIP-APCI: [M+H]+
Cyanox 2246	Antioxidant	119-47-1	C23H32O2	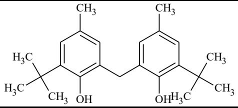	10	LC-ESI: [M+H]+
Cyanox 1790	Antioxidant	40601-76-1	C42H57N3O6	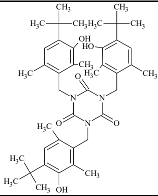	6	LC-ESI: [M+Na]+, [M-H]-<br>LC-APCI: [M+H]+, [M-H$_2$]-
Cyanox LTDP	Antioxidant	123-28-4	C30H58O4S	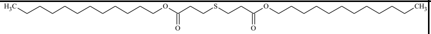	17	LC-ESI: [M+Na]+, [M-H]-<br>LC-APCI: [M+H]+
Naugard BHT	Antioxidant	128-37-0	C15H24O	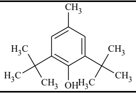	14	LC-ESI: [M-H]-<br>LC-APCI: [M-H]-<br>DIP-APCI: [M-H]-
Ultranox 626 phosphite	Antioxidant	26741-53-7	C33H50O6P2	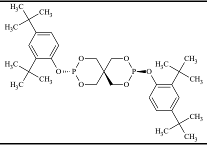	3	LC-APCI: [M+H]+
Dibutyl phthalate (DBP)	Plasticizer- phthalate[b]	84-74-2	C16H22O4	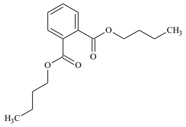	11	LC-ESI: [M+Na]+<br>LC-APCI: [M+H]+<br>DIP-APCI: [M+H]+
Diethyl phthalate (DEP)	Plasticizer- phthalate	84-66-2	C12H14O4	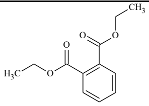	1	LC-ESI: [M+Na]+<br>LC-APCI: [M+H]+<br>DIP-APCI: [M+H]+
Dipentyl phthalate (DPP)	Plasticizer- phthalate[b]	131-18-0	C18H26O4	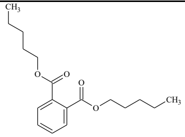	7	LC-ESI: [M+Na]+<br>LC-APCI: [M+H]+<br>DIP-APCI: [M+H]+
Di-isononyl phthalate (DINP)	Plasticizer- phthalate[b]	28553-12-0	C26H42O4	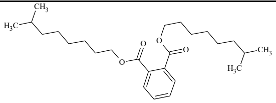	5	LC-ESI: [M+Na]+<br>LC-APCI: [M+H]+<br>DIP-APCI: [M+H]+

Bis(2-ethylhexyl) phthalate (DEHP)	Plasticizer- phthalate[b]	117-81-7	C24H38O4	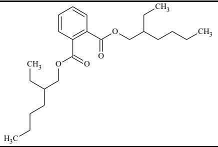	20	LC-ESI: [M+Na]+<br>LC-APCI: [M+H]+<br>DIP-APCI: [M+H]+
Di-n-hexyl phthalate (DnHP)	Plasticizer- phthalate[b]	84-75-3	C20H30O4	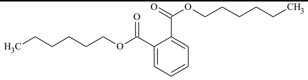	3	LC-ESI: [M+Na]+<br>LC-APCI: [M+H]+<br>DIP-APCI: [M+H]+
Benzyl butyl phthalate (BBP)	Plasticizer- phthalate	85-68-7	C19H20O4	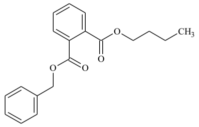	7	LC-ESI: [M+Na]+<br>LC-APCI: [M+H]+<br>DIP-APCI: [M+H]+
Butyl cyclohexyl phthalate (BCP)	Plasticizer- phthalate[b]	84-64-0	C18H24O4	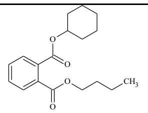	2	LC-ESI: [M+Na]+<br>LC-APCI: [M+H]+
Butyl decyl phthalate (BDP)	Plasticizer- phthalate[b]	89-19-0	C22H34O4	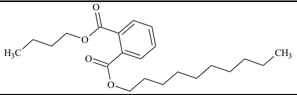	1	LC-APCI: [M+H]+
Di-n-propyl phthalate (DnPP)	Plasticizer- phthalate[b]	131-16-8	C14H18O4	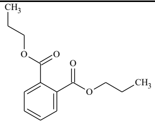	5	LC-APCI: [M+H]+
Diundecyl phthalate (DUP)	Plasticizer- phthalate[b]	3648-20-2	C30H50O4	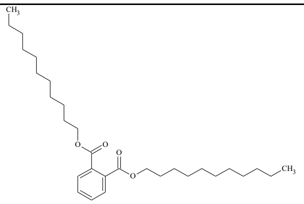	5	LC-ESI: [M+Na]+<br>LC-APCI: [M+H]+
UV absorber-120	Light stabilizer	4221-80-1	C29H42O3	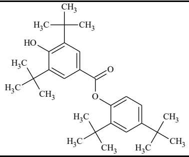	1	LC-ESI: [M-H]-<br>DIP-APCI: [M-H]-
Cyasorb 2908	Light stabilizer	67845-93-6	C31H54O3	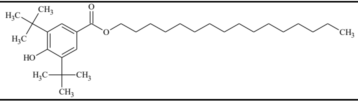	10	LC-ESI: [M+Na]+<br>LC-APCI: [M+H]+<br>DIP-APCI: [M+H]+
Tinuvin 234	Light stabilizer	70321-86-7	C30H29N3O	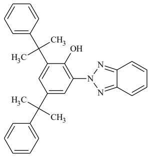	4	LC-ESI: [M-H]-<br>LC-APCI: [M+H]+, [M-H]-
Tinuvin 326	Light stabilizer	3896-11-5	C17H18ClN3O	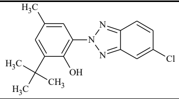	7	LC-ESI: [M-H]-<br>LC-APCI: [M+H]+, [M-H]-<br>DIP-APCI: [M+H]+, [M-H]-

Tinuvin 327	Light stabilizer	3864-99-1	C20H24ClN3O	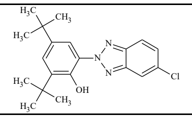	10	LC-ESI: [M-H]-<br>LC-APCI: [M+H]+, [M-H]-<br>DIP-APCI: [M+H]+, [M-H]-
Tinuvin 328	Light stabilizer	25973-55-1	C22H29N3O	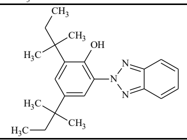	7	LC-ESI: [M+H]+, [M-H]-<br>LC-APCI: [M+H]+, [M-H]-<br>DIP-APCI: [M+H]+, [M-H]-
Tinuvin 329	Light stabilizer	3147-75-9	C20H25N3O	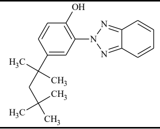	10	LC-ESI: [M-H]-<br>LC-APCI: [M+H]+, [M-H]-<br>DIP-APCI: [M+H]+, [M-H]-
Tinuvin 360	Light stabilizer	103597-45-1	C41H50N6O2	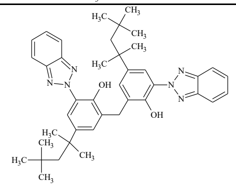	4	LC-ESI: [M-H]-<br>DIP-APCI: [M+H]+, [M-H]-
Tinuvin PED	Light stabilizer	2440-22-4	C13H11N3O	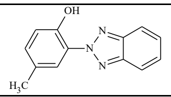	11	LC-ESI: [M-H]-, [M+H]+,<br>LC-APCI: [M+H]+, [M-H]-<br>DIP-APCI: [M+H]+, [M-H]-
Tinuvin 770	Light stabilizer	52829-07-9	C28H52N2O4	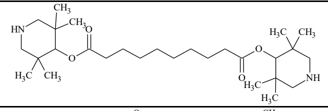	8	LC-ESI: [M+H]+,<br>LC-APCI: [M+H]+<br>DIP-APCI: [M+H]+
Uvinul 3008	Light stabilizer	1843-05-6	C21H26O3	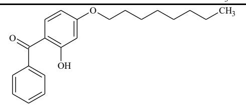	5	LC-ESI: [M-H]-<br>LC-APCI: [M-H]-, [M+H]+<br>DIP-APCI: [M-H]-
Diphenyl phosphate (DPHP)	Phosphorus flame retardant/plasticizer (impurity/byproduct/degradation product)		C12H11O4P	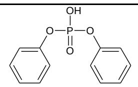	10	LC-ESI: [M-H]-<br>(DIP-APCI: most probably false positives)
Dihydroxy-triphenyl phosphate	Phosphorus flame retardant/plasticizer (impurity/byproduct/degradation product)		C18H15O6P	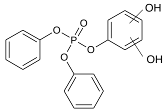<br>(the position of the –OH groups is unknown)	9	LC-ESI: [M-H]-<br>LC-APCI: [M-H]-<br>DIP-APCI: [M-H]-
Meta-hydroxy-triphenyl phosphate	Phosphorus flame retardant/plasticizer (impurity/byproduct/degradation product)		C18H15O5P	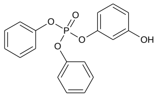	13	LC-ESI: [M-H]-, [M+Na]+,<br>LC-APCI: [M-H]-, [M+H]+,[M+CH3OH+H]+<br>DIP-APCI: [M-H]-, [M+H]+
Triphenyl phosphate (TPHP)	Phosphorus flame retardant/plasticizer	115-86-6	C18H15O4P	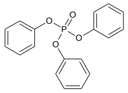	19	LC-ESI: [M+H]+, [M+Na]+,<br>LC-APCI: [M+H]+, [M+CH3OH+H]+<br>DIP-APCI: [M+H]+

Resorcinol bis(diphenyl phosphate) with a loss of a phenyl group<br>RDP-[Phe]	Phosphorus flame retardant/plasticizer (impurity/byproduct/ degradation product)		C24H20O8P2	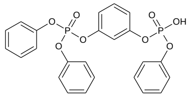	7	LC-ESI: [M-H]-<br>(DIP-APCI: most probably false positives)
Hydroxy-Resorcinol bis(diphenyl phosphate)	Phosphorus flame retardant/plasticizer (impurity/byproduct/degradation product)		C30H24O9P2	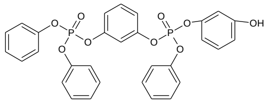<br>(the most probable position of the –OH group is *meta*- as breakdown product coming from RDP oligomers)	6	LC-ESI: [M-H]-, [M+Na]+,<br>LC-APCI: [M-H]-, [M+H]+, [M+CH3OH+H]+,<br>DIP-APCI: [M-H]-
Resorcinol bis(diphenyl phosphate) (RDP)	Phosphorus flame retardant/plasticizer	57583-54-7	C30H24O8P2	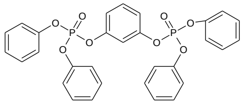	13	LC-ESI: [M-H]-, [M+Na]+<br>LC-APCI: [M+H]+, [M+CH3OH+H]+,<br>DIP-APCI: [M+H]+, [M+CH3OH+H]+,
RDP dimer [RDP-n2]	Phosphorus flame retardant/plasticizer-oligomer		C42H33O12P3	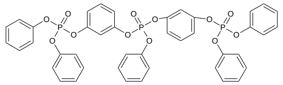	12	LC-ESI: [M+Na]+, $[M+CH_3CNNH_3+H]^+$<br>LC-APCI: [M+H]+<br>DIP-APCI: [M+H]+
RDP trimer [RDP-n3]	Phosphorus flame retardant/plasticizer-oligomer		C54H42O16P4	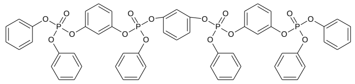	2	LC-ESI: [M+Na]+, $[M+CH_3CNNH_3+H]^+$<br>LC-APCI: [M+H]+<br>DIP-APCI: [M+H]+
Bisphenol A bis(diphenyl phosphate) (BDP)	Phosphorus flame retardant/plasticizer	5945-33-5	C39H34O8P2	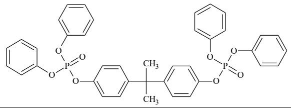	12	LC-ESI: [M+H]+<br>LC-APCI: [M+H]+, [M+CH3OH+H]+<br>DIP-APCI: [M+H]+
Tris (isopropylphenyl) phosphate (TIPP)	Phosphorus flame retardant/plasticizer	26967-76-0	C27H33O4P	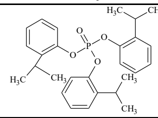	1	LC-ESI: [M+Na]+<br>LC-APCI: [M+H]+<br>DIP-APCI: [M+H]+
2-Ethylhexyl diphenyl phosphate (EHDP)	Phosphorus flame retardant/plasticizer	1241-94-7	C20H27O4P	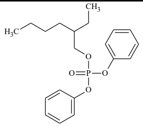	6	LC-APCI: [M+H]+, [M-C8H17+H2]+<br>DIP-APCI: [M+H]+ [M-C8H17+H2]+
Tris(2-butoxyethyl) phosphate (TBEP)	Phosphorus flame retardant/plasticizer	78-51-3	C18H39O7P	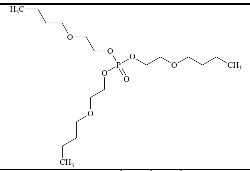	8	LC-ESI: [M+Na]+<br>LC-APCI: [M+H]+<br>DIP-APCI: [M+H]+
Tris(2-ethylhexyl) phosphate (TEHP)	Phosphorus flame retardant/plasticizer	78-42-2	C24H51O4P	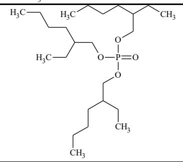	3	LC-ESI: [M+Na]+<br>LC-APCI: [M+H]+

di-tert-butylphenyl phosphate (dtBPP)	Phosphorus flame retardant/plasticizer	65652-41-7	C26H31O4P	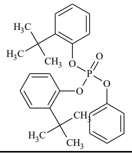	6	LC-ESI: [M+Na]+<br>LC-APCI: [M+H]+<br>DIP-APCI: [M+H]+
tert-Butylphenyl diphenyl phosphate (BPDP)	Phosphorus flame retardant/plasticizer	56803-37-3	C22H23O4P	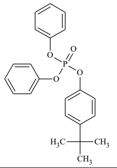	6	LC-ESI: [M+Na]+<br>LC-APCI: [M+H]+<br>DIP-APCI: [M+H]+
Isodecyl diphenyl phosphate (IDPP)	Phosphorus flame retardant/plasticizer	29761-21-5	C22H31O4P	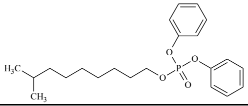	3	LC-ESI: [M+H]+, [M+Na]+<br>LC-APCI: [M+H]+
Tri-p-tert-butylphenyl-phosphate (ttBPP)	Phosphorus flame retardant/plasticizer	78-33-1	C30H39O4P	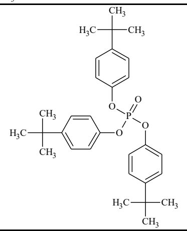	2	LC-ESI: [M+Na]+<br>LC-APCI: [M+H]+
Cresyl diphenyl phosphate (CDP)	Phosphorus flame retardant/plasticizer	26444-49-5	C19H17O4P	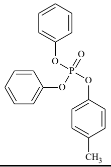	6	LC-ESI: [M+Na]+<br>LC-APCI: [M+H]+
Tricresyl phosphate isomers (TCP o TMPP)	Phosphorus flame retardant/plasticizer	62974-06-5	C21H21O4P	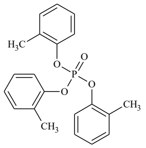	7	LC-ESI: [M+Na]+<br>LC-APCI: [M+H]+<br>DIP-APCI: [M+H]+
Tris (1-chloro-2-propyl) phosphate (TCPP)	Phosphorus flame retardant/plasticizer	13674-84-5	C9H18Cl3O4P	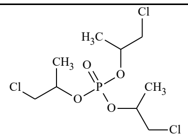	1	LC-ESI: [M+H]+, [M+Na]+<br>LC-APCI: [M+H]+
Dioctyl sebacate (DCS)	plasticizer-non-phthalate	122-62-3	C26H50O4	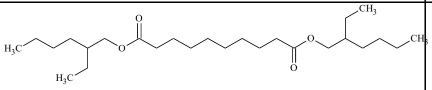	3	LC-ESI: [M+Na]+<br>LC-APCI: [M+H]+
Bisphenol A (BPA)	plasticizer-non-phthalate	80-05-7	C15H16O2	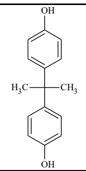	21	LC-ESI: [M-H]-<br>LC-APCI: [M-H]-<br>DIP-APCI: [M-H]-

2,4,6-Tribromophenol	Intermediate in the preparation of flame retardants	118-79-6	C6H3Br3O	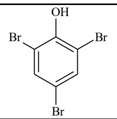	8	LC-ESI: [M-H]-<br>LC-APCI: [M-H]-<br>DIP-APCI: [M-H]-
Dibromo-bisphenol A	Brominated flame retardant (impurity/byproduct/ degradation product)		C15H14Br2O2	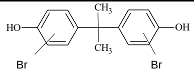<br>(the position of the Br atoms is unknown)	6	LC-ESI: [M-H]-<br>LC-APCI: [M-H]-<br>DIP-APCI: [M-H]-
Tribromo-bisphenol A	Brominated flame retardant (impurity/byproduct/ degradation product)		C15H13Br3O2	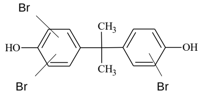<br>(the position of the Br atoms is unknown)	8	LC-ESI: [M-H]-<br>LC-APCI: [M-H]-<br>DIP-APCI: [M-H]-
Tetrabromobisphenol A (TBBPA)	Brominated flame retardant	79-94-7	C15H12Br4O2	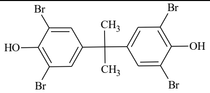	11	LC-ESI: [M-H]-<br>LC-APCI: [M-H]-, [M-Br+O]-<br>DIP-APCI: [M-H]-, [M-Br+O]-
TBBPA mono(glycidyl ether) (TBBPA-MGE)	Brominated flame retardant (impurity/byproduct)		C18H16Br4O3	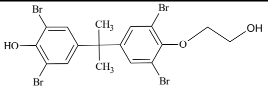	7	LC-ESI: [M-H]-<br>LC-APCI: [M-H]-<br>DIP-APCI: [M-H]-
TBBPA mono(2-hydroxyethyl ether) (TBBPA-MHEE)	Brominated flame retardant (impurity/byproduct)		C17H16Br4O3	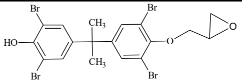	7	LC-ESI: [M-H]-<br>LC-APCI: [M-H]-<br>DIP-APCI: [M-H]-
Heptabromodiphenyl ether isomers	Brominated flame retardant	207122-16-5<br>117948-63-7<br>189084-68-2<br>446255-30-7	C12H3Br7O	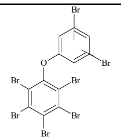	5	LC-APCI: [M-Br+O]-<br>DIP-APCI: [M-Br+O]-
Octabromodiphenyl ether isomers	Brominated flame retardant	446255-39-6<br>117964-21-3<br>67797-09-5<br>337513-72-1<br>446255-54-5<br>446255-56-7	C12H2Br8O	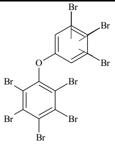	5	LC-APCI: [M-Br+O]-<br>DIP-APCI: [M-Br+O]-
Nonabromodiphenyl ether isomers	Brominated flame retardant	63936-56-1<br>437701-79-6<br>437701-78-5	C12HBr9O	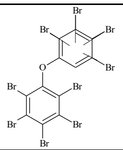	5	LC-APCI: [M-Br+O]-<br>DIP-APCI: [M-Br+O]-
DecaBDE	Brominated flame retardant	1163-19-5	C12Br10O	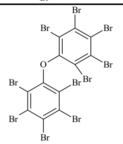	5	LC-APCI: [M-Br+O]-<br>DIP-APCI: [M-Br+O]-

Tris(2,4,6-tribromophenoxy)-1,3,5-triazine (TTBP-TAZ)	Brominated flame retardant	25713-60-4	C21H6Br9N3O3	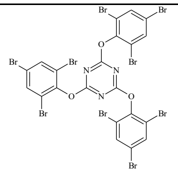	8	LC-APCI: [M+H]+, [M-C6H2Br3-H]-<br>LC-APCI: [M+H]+, [M-C6H2Br3-H]-<br>DIP-APCI: [M+H]+, [M-C6H2Br3-H]-
1,2-Bis(2,4,6-Tribromophenoxy)ethane (BTBPE)	Brominated flame retardant	37853-59-1	C14H8Br6O2	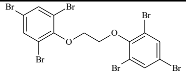	2	LC-APCI: Fragment [C6Br3H2O]-<br>DIP-APCI: Fragment [C6Br3H2O]-
Decabromodiphenyl ethane (DBDPE)	Brominated flame retardant	84852-53-9	C14H4Br10	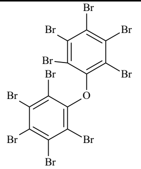	5	LC-APCI: [M-Br+O]-<br>DIP-APCI: [M-Br+O]-

[a] Only those confirmed by LC; [b] Structural isomers sharing the same molecular formula are frequent among phthalates, e.g. n-alkyl chains and iso-alkyl chains, the most common isomer in the literature was considered as the most probable and it is reported in this study.

Table S-3. Predicted toxicological concern and biodegradability for the identified impurities, degradation products and oligomers of flame retardants.

Compound	Chemical structure	Toxicological concern[a]	Biodegradability[b]	Carcinogenicity[c]	Mutagenicity[c,d]
Diphenyl phosphate (DPHP)	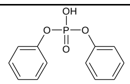	Low	Persistent	No alerts	No alerts
Dihydroxy-triphenyl phosphate	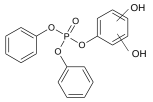	High	Persistent	No alerts	No alerts
Meta-hydroxy-triphenyl phosphate	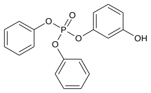	High	Persistent	No alerts	No alerts
Resorcinol bis(diphenyl phosphate) with a loss of a phenyl group RDP-[Phe]	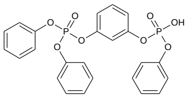	High	Persistent	No alerts	No alerts
Dibromo-bisphenol A	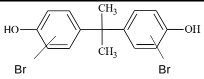	High	Persistent	No alerts	No alerts
Tribromo-bisphenol A	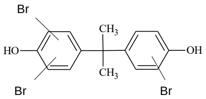	High	Persistent	No alerts	No alerts
TBBPA mono(glycidyl ether) (TBBPA-MGE)	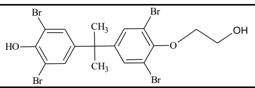	High	Persistent	No alerts	No alerts
TBBPA mono(2-hydroxyethyl ether) (TBBPA-MHEE)	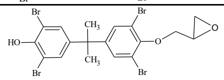	High	Persistent	Structural alert for genotoxic carcinogenicity	Structural alert for S. typhimurium mutagenicity

Prediction calculated with Toxtree (http://toxtree.sourceforge.net/), decision trees: [a]Cramer rules with extensions; [b]START biodegradability; [c]carcinogenicity (genotox and nongenotox) and mutagenicity rulebase by ISS ; [d]*in-vitro* mutagenicity (Ames test) alerts by ISS
